# Evaluating Co-Ensiling Strategies to Valorise Duckweed as a Sustainable Feed Ingredient

**DOI:** 10.3390/plants15121865

**Published:** 2026-06-16

**Authors:** Marie Lambert, Eva Wambacq, Reindert Devlamynck, Marcella Fernandes de Souza, Pieter Vermeir, Katleen Raes, Mia Eeckhout, Erik Meers

**Affiliations:** 1 Laboratory for Bioresource Recovery, Department of Green Chemistry and Technology, Faculty of Bioscience Engineering, Ghent University, Coupure Links 653, 9000 Ghent, Belgium; marcella.fernandesdesouza@ugent.be; 2 Research Centre AgroFoodNature, School of Bioscience and Industrial Technology, University of Applied Sciences and Arts, Valentin Vaerwyckweg 1, 9000 Ghent, Belgium; eva.wambacq@hogent.be (E.W.);; 3 Provincial Research and Advice Centre for Agriculture and Horticulture (Inagro vzw), Ieperseweg 87, 8800 Roeselare-Beitem, Belgium; reindert.devlamynck@inagro.be; 4 Laboratory for Chemical Analysis (LCA), Department of Green Chemistry and Technology, Faculty of Bioscience Engineering, Ghent University, Valentin Vaerwyckweg 1, 9000 Ghent, Belgium; 5 Research Unit VEG-i-TEC, Department of Food Technology, Safety and Health, Campus Kortrijk, Ghent University, St-Martems Latemlaan 2B, 8500 Kortrijk, Belgium; 6 Research Unit of Cereal and Feed Technology, Department of Food Technology, Safety and Health, Faculty of Bioscience Engineering, Ghent University, 9000 Ghent, Belgium

**Keywords:** ensiling, duckweed, co-silage crops, feed preservation, corn silage, grass silage, beet pulp

## Abstract

Duckweed (Lemnaceae) is a promising alternative feed crop, particularly in regions with nutrient surpluses and protein deficits, as it grows efficiently on nutrient-rich agricultural wastewater and provides protein-rich biomass. However, its high moisture content and rapid post-harvest spoilage pose major storage challenges. This study evaluated (co-)ensiling as a cost-effective preservation strategy for duckweed. Three separate experiments were conducted to assess the ensilability of duckweed alone and in combination with various agricultural co-substrates and additives, including corn silage, beet pulp, grass silage, hemp shives, hay, molasses, sun-dried duckweed and CaCO_3_. Duckweed alone could not be successfully ensiled due to excessive moisture, resulting in poor acidification and high levels of undesirable fermentation products. During the long-term co-ensiling test, a duckweed–corn silage mixture containing 29% fresh duckweed and 71% corn silage showed the most stable fermentation profile, with low pH, limited fermentation losses, and no detectable butyric acid. A duckweed–grass silage mixture containing 51% fresh duckweed and 49% grass silage allowed higher duckweed inclusion and retained the highest level of apparent pepsin-digestible protein after storage, but showed elevated acetic acid and ethanol concentrations. A duckweed–beet pulp mixture containing 74% fresh duckweed and 26% beet pulp enabled the highest duckweed inclusion rate, but showed signs of clostridial fermentation, likely due to excess moisture. Microbiological analysis of this beet pulp mixture showed reduced *Enterobacteriaceae* after ensiling, but also increased clostridial counts. Oxalic acid concentrations were low in all duckweed-based silages, with the largest reduction observed in the duckweed–grass mixture. Overall, the results show that duckweed co-ensiling is feasible but highly dependent on co-substrate selection and moisture control. Further formulation optimisation is required, particularly for high-duckweed mixtures, to reduce the risk of clostridial fermentation and improve practical applicability as a storable feed ingredient.

## 1. Introduction

In several regions of Europe with high livestock density, such as Flanders (Belgium), agriculture faces two major challenges: a surplus of nutrients (mainly nitrogen and phosphorus) on the one hand and a high dependency on imports of high-protein feedstocks on the other hand. A potential sustainable solution is to grow duckweed (Lemnaceae) on nutrient-rich agricultural waste streams and use the resulting biomass as a protein source in animal diets [1,2,3]. Duckweed is highly effective at removing nutrients from water and grows quickly, making it a valuable tool for water treatment [4]. Additionally, its biomass has a high protein content (30–45% on dry matter), along with beneficial (micro)nutrients [1,2,5]. Because duckweed grows on the water surface, it can be harvested mechanically in controlled cultivation systems and can be installed across a variety of agricultural or non-arable sites.

Despite its environmental and agronomic advantages, duckweed also has important practical limitations as a feed crop. Its high moisture content, typically over 90%, poses major challenges for transport, storage, and preservation. In addition, its low dry matter content means that the nutritional value per unit fresh weight is relatively diluted. While fresh duckweed can be fed directly to animals, this requires close alignment of harvest and feeding schedules. Moreover, when water temperatures drop below 8 °C or exceed 30–32 °C for a prolonged period, duckweed growth slows down or stops, preventing year-round production in many climates [6]. In Flanders, for example, this results in a growing season of approximately 175 days in outdoor conditions [2].

Ensiling duckweed could be a low-cost and scalable solution to overcome this preservation challenge. During this anaerobic fermentation process, naturally present (or inoculated) lactic acid bacteria (LAB) convert soluble carbohydrates into organic acids, primarily lactic acid. This process reduces the pH, inhibits spoilage, and maximally preserves the nutritional quality of the biomass during a prolonged period [7]. However, successful ensiling requires sufficient dry matter content, fermentable carbohydrates, and rapid acidification. Duckweed is therefore intrinsically challenging as an ensiling substrate because its high moisture content and low water-soluble carbohydrate availability can limit lactic acid production and increase the risk of undesirable fermentations, including clostridial fermentation.

Only limited research has been conducted on the feasibility of ensiling duckweed. In a study by Abdel-Rahman et al. (2021), mixtures of duckweed with rice straw and molasses were ensiled, showing promising preservation results [8]. They also reported positive effects on digestibility and nutritive value compared to corn silage in a feeding trial with Barki lambs. Yet, as rice straw is not commonly available in all countries, it is relevant to investigate alternative co-substrates for ensiling.

Another study, conducted in the Netherlands by Hoving et al. (2011), ensiled duckweed alone or mixed with acid, molasses (with or without LAB inoculant), dry duckweed pulp, or corn silage, and concluded that duckweed cannot be preserved successfully without additives [9]. Their tests also showed that the addition of molasses resulted in improved silage preservation. In addition, they evaluated the practical ensiling process and found that conventional methods for forage ensiling are not suitable for duckweed due to its low structural content, which makes it difficult to wrap. Instead, they recommended airtight containers or vertical silos to ensure good preservation and reduce losses. However, their work did not assess the effect of co-substrates other than corn on the silage quality, nor were the (anti-)nutritional effects of ensiling explored.

The goal of this research was to develop effective strategies for preserving duckweed through ensiling. In particular, the study aimed to identify suitable ensiling co-substrates and the effect of molasses addition during silage fermentation while offering flexibility in function of seasonal and regional availability. Silage quality of the best-performing conditions was evaluated based on dry matter content, fermentation losses, pH, ammonia-nitrogen fraction, and concentrations of key fermentation products such as lactic acid, acetic acid, butyric acid, and ethanol. To better understand the impact of ensiling on nutritional value, additional assessments of selected ensiling mixtures included oxalic acid content, pepsin digestibility, and microbial composition before and after storage.

## 2. Materials and Methods

### 2.1. Duckweed Species and Cultivation

Ensiling experiments were conducted using *Lemna* harvested from different duckweed wastewater treatment ponds, and ensiling always took place on the same day as harvesting. The first experiment utilized *Lemna minor* harvested from a duckweed pond fertilised with pig manure effluent (51.1302° N, 3.0512° E) in September 2019. A description of the cultivation conditions can be found in earlier published work by Devlamynck et al. [1]. For the second and third experiments, Lemna × japonica 6580 (originally obtained as *L. minor* 6580 from the Landolt collection, but later revealed to be *L.* × *japonica*) was harvested from a duckweed pond (35 m^2^) filled and daily fertilised with aquaculture effluent (50.9036° N, 3.1262° E) in September 2024.

### 2.2. Ensiling Experiments

To evaluate the feasibility of ensiling duckweed, three separate experiments were conducted and are summarized in Table 1.

In the first experiment, the objective was to assess whether fresh duckweed, without additives or ensiling co-substrates, could be successfully ensiled. The main purpose of adding a co-substrate was to increase the dry matter content of the mixture and thereby improve its ensilability. As the duckweed was cultivated on medium fertilised with pig manure effluent, which was visibly richer in suspended organic material than the aquaculture effluent used in the subsequent tests, the effect of a simple washing step on the measured silage quality parameters was also investigated. For the washed treatments (T3 and T5), duckweed was divided into smaller portions and rinsed three times until the rinse water appeared visually clear. The purpose of this washing step was to remove residual cultivation medium before ensiling. No microbiological or compositional analyses were performed directly before and after washing; therefore, the washing treatment was evaluated only through the final silage quality parameters. In both subsequent experiments, duckweed grown on aquaculture effluent was used, and washing was not included due to the cleaner nature of this medium.

The second experiment screened various silage mixtures based on pH reduction over a 9-day ensiling period. The third experiment evaluated four selected mixtures in terms of storage stability and silage quality after an ensiling period of 102 days.

Unless duckweed was ensiled alone or in combination with molasses, a maximum amount of duckweed was combined with a co-substrate to obtain a final mixture with a dry matter content of around 30%, based on the measured dry matter content of the separate feedstuffs. However, due to the time gap between dry matter determination and mixing of the co-substrates, the final dry matter content occasionally deviated from the 30% target, as can be seen in Table 1. In the second experiment, treatment T9 intentionally included a higher proportion of duckweed, resulting in a dry matter content of approximately 20%.

For both Tests I and III, conducted in quadruplicates, the starting materials were manually homogenised and uniformly pressed into 2.75 L microsilos containing approximately 2 kg of ensiling mixture, following the same approach as described by Nikodinoska et al. (2024) [10]. The mixtures were introduced using an extension tube. After partial filling, the material was pressed to reduce the volume, after which the remaining material was added and pressed again. The microsilos were weighed empty, after filling, and after storage.

In Test I, conventional microsilos were used for T1, T4, and T5, whereas microsilos with effluent juice collection were used for the high-moisture duckweed-only treatments T2 and T3. Effluent juice released during ensiling was collected at the bottom of these microsilos via a tube connected to a plastic bag. These collected juices were not analysed and are therefore not further discussed in this study. In Test III, conventional microsilos were used for all the treatments, as the mixtures were formulated to reach a sufficiently high dry matter content to avoid effluent losses. In Tests II and III, effluent release was not collected or quantified separately.

The microsilos were airtight at the bottom and lid and equipped with a Bunsen valve to allow fermentation gases to escape whilst maintaining anaerobic conditions in the microsilos. The silo density was calculated and is reported in Appendix A Table A1. Except for the duckweed-only treatments in Test I, all the treatments were within the range of values that can be encountered in practical silages.

The microsilos were stored in the barn of the experimental farm in Bottelare (50.9620° N, 3.7610° E) during the silage conservation period. The barn temperature during the experiments ranged between approximately 12 and 22 °C. In Test I, the microsilos were opened after 42 days, while in Test III, the microsilos were opened after 102 days. After opening, the central part of the silo was collected and manually mixed, sampled, and analysed.

For Test II (in duplicates), smaller volumes of 300 g fresh weight were ensiled in vacuum bags that were stored at room temperature (approximately 20 °C). For each treatment, 8 bags were prepared to open in duplicate at 1, 2, 4, and 9 silage-days.

For each test, one bulk mixture was prepared per treatment, with each treatment indicated by a T-code (see Table 1). After manual homogenisation, a representative subsample was taken from this bulk mixture for the analyses of the pre-ensiling material. The remaining material was then divided over the replicate silos or bags: four microsilos for Tests I and III, and duplicate vacuum bags for each opening time in Test II. After ensiling, each silo or bag was opened separately, and subsamples were taken from each replicate according to the requirements of the different analyses. Samples were stored at −18 °C until analysis when immediate analysis was not possible.

### 2.3. Silage Quality Evaluation

#### 2.3.1. Analyses on the Starting Materials

Dry matter content of duckweed, co-substrates, and the prepared mixtures was determined by drying in a Weiss Technik VTU 100/150 250 °C drying oven (Weiss Technik, Reiskirchen, Germany) at 65 °C until constant weight. In Test II (Table 1), only the dry matter content of the separate feedstuffs before ensiling was determined. Crude ash was determined after incineration at 550 ± 25 °C (according to ISO 5984 [11]), and crude protein was determined following ISO 16634-1 [12] (Kjeldahl, factor 6.25). Water Soluble Carbohydrates (WSC) were determined according to Luff-Schoorl’s method (NEN3571) [13], while buffering capacity was determined by titration with 0.1N HCl to pH 4.0 (modified method based on Jasaitis et al. (1987)) and recalculated to lactic acid [14].

In test III, the corn silage used as co-substrate had a particle size of approximately 6–10 mm, while the grass silage consisted of prewilted grass silage with a particle size of approximately 6–8 cm. Beet pulp was used as dried pellets. The dry matter contents of the co-substrates are reported in Appendix A Table A2.

#### 2.3.2. Analyses on the Ensiled Mixtures

All micro-silos were weighed empty, just after filling, and at silo opening to enable the calculation of the fermentation losses on a fresh weight basis. Fermentation losses were presented on a fresh weight basis to avoid additional uncertainty associated with converting these values to a dry matter basis, which would require correction for volatile losses during drying. In Test I, the fermentation loss was monitored weekly. In Test III, the fermentation loss was only measured at the end of the 102-day ensiling period. Dry matter content was determined by air drying at 65 °C until constant weight and corrected for loss of volatiles during drying according to Weissbach & Kuhla (1995), following the same method as the research on duckweed ensiling done by Hoving et al. (2011) [9,15]. Total nitrogen was determined using the Dumas method according to ISO 16634-1 [12], and crude protein was calculated using a conversion factor of 6.25. Ammoniacal nitrogen was determined in a water extract by steam distillation. The ammonia fraction was calculated as the ratio of ammonia nitrogen to total nitrogen [16]. The pH was determined on an aqueous extract according to De Boever et al. [17]. Lactic acid, acetic acid, butyric acid, and propionic acid were determined on the same aqueous extract by HPLC according to Ohmomo et al. (1993) [18]. Oxalic acid was determined by HPLC (Agilent 1260 Infinity II with column Hi-Plex H, 300 × 7.7 mm, 8 mm, Agilent, Santa Clara, CA, USA) after extraction in 5 mM H_2_SO_4_ in a 1:20 mass solvent ratio, with mixing the sample for 30 s at 10,000 rpm at room temperature. Ethanol was determined on an aqueous extract by NIR absorption according to EBC 9.2.6. WSC was determined by Luff-Schoorl’s method (NEN 3571) [13]. The ratio of lactic acid to acetic acid was calculated, as well as the sum of fermentation products (i.e., sum of lactic acid, acetic acid, propionic acid, butyric acid, and ethanol). In Test II (Table 1), only the pH before and after ensiling was measured in an aqueous extract, following the method by Müller & Amend (1997) [19].

#### 2.3.3. Pepsin Digestibility

In Test III (Table 1), in addition to the crude protein content before and after ensiling, the pepsin digestibility of fresh duckweed and the ensiled mixtures before and after ensiling was determined. Fresh duckweed, as well as the three mixed samples of starting materials, were analysed in duplicate. The mixed samples obtained after ensiling, which were prepared in experimental quadruplicate, were analysed in singlet. The pepsin digestibility was assessed following the first digestion step of the Megazyme K-PDCAAS in vitro protein digestibility protocol, as also applied in other in vitro digestibility studies [20]. However, in the present study, only the pepsin digestion step was used to estimate the apparent pepsin-soluble protein fraction; the subsequent trypsin/chymotrypsin digestion and PDCAAS calculation were not performed.

Ground, frozen samples were stored below −10 °C and thoroughly homogenized prior to weighing. For each sample, 0.5 g was weighed and suspended in 19 mL of 0.06 N HCl in a centrifuge tube. The mixture was thoroughly vortexed and incubated at 37 °C for 30 min with shaking at 150 rpm. Subsequently, 1 mL of pepsin solution (1 mg/mL, pepsin obtained from the Megazyme K-PDCAAS kit, Bray, Ireland) was added, and the samples were vortexed. Next, the samples were further incubated for 1 h under the same conditions (37 °C, 150 rpm).

Following incubation, enzymatic activity was halted by placing the samples in a boiling water bath for 10 min. The samples were then vortexed and centrifuged (Frontier 5000 series multi Pro, OHAUS, Parsippany, New Jersey, USA) at 4500 rpm for 15 min. The digestible protein content in the resulting supernatant was quantified using the Lowry method, with Bovine Serum Albumin (BSA) as the standard. Casein was included as an internal standard throughout the extraction process. The pepsin-digestible protein content was then compared to the crude protein content before pepsin digestion, measured according to ISO 16634-1 (factor 6.25) [12].

#### 2.3.4. Microbiology

In Test III (Table 1), different microbiological analyses were done on the fresh duckweed used to prepare the mixtures and on the ensiled mixture with beet pulp (T2) obtained after an ensiling period of 102 days. The obtained data were then compared to feed standards. The microbiological analyses were outsourced to ILVO (Melle, Belgium). A list of the microbiological analyses performed with their specific unit and the used method is presented in Appendix A, Table A3.

#### 2.3.5. Data Processing and Statistics

Data processing and figure preparation were performed in Microsoft Excel, while statistical analyses were performed in RStudio (version 4.3.1). Tests I and III were conducted in quadruplicate, whereas Test II was conducted in duplicate as an exploratory screening test for the selection of treatments for Test III. Mean values and standard deviations were calculated for all the measured parameters. No inferential statistics were applied to Test II because of the limited number of replicates and its screening purpose.

For Tests I and III, statistical differences between treatments were assessed at *p* < 0.05. Normality of residuals was checked using Q-Q plots and the Shapiro–Wilk test, and homogeneity of variance was assessed using box plots and the modified Levene’s test. When these assumptions were met, one-way ANOVA followed by Tukey’s post hoc test was used. When assumptions were not met, the Kruskal–Wallis test followed by Dunn’s post hoc test was applied. For oxalic acid, total protein, and pepsin-digestible protein in Test III, statistical differences were assessed only between the three ensiled treatments, which were available as four biological replicates. These differences were tested using one-way ANOVA followed by Tukey’s post hoc test, as the assumptions of normality and homogeneity of variance were met. The pre-ensiling mixtures consisted of one composite sample per treatment, prepared in bulk before being divided over four replicate silos. Therefore, pre-ensiling values were not included in the statistical analysis but are shown in the figures for descriptive comparison.

## 3. Results and Discussion

The three ensiling tests described here were designed as a sequential, practice-oriented approach to develop co-ensiling strategies for duckweed, rather than as a fully comparable factorial experiment. As described in the Materials and Methods, the duckweed source and some co-substrates differed between tests. These differences reflect the practical nature of the experiments, which were conducted over an extended period using the biomass and co-substrates available at each time point. However, they also limit direct quantitative comparison between tests.

Although the duckweed batches had relatively comparable dry matter contents, ranging from approximately 6 to 9% DM, differences in cultivation medium, biomass composition, associated microbiota, and co-substrate type or condition may have affected the ensiling outcome. This is relevant because silage quality is influenced by crop composition and ensiling characteristics, including dry matter content, fermentable carbohydrates, and substrate structure [21]. Consequently, conclusions are drawn within each test, while the outcome of each test was used to guide the design of the next step in the sequential development of co-ensiling strategies.

### 3.1. Duckweed Is Unsuitable as a Sole Substrate for Effective Silage Production, but Co-Ensiling Is a Promising Strategy

**Figure 1:**
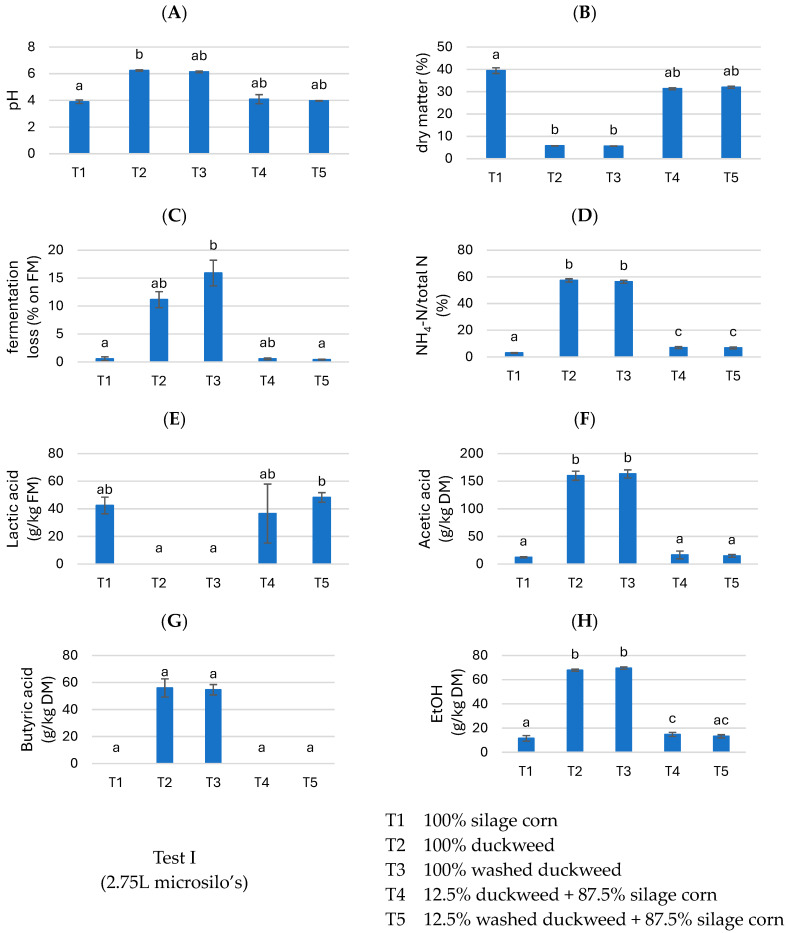
pH (**A**), dry matter content (**B**), total fermentation loss (on fresh weight) (**C**), ammonia fraction (**D**), lactic acid (**E**), acetic acid (**F**), butyric acid (**G**), and ethanol concentration (**H**) on dry matter basis measured on 5 different silage mixtures after a 42-day ensiling period (Test I, Table 1 and as presented in the legend). Mean values are presented with standard deviations, based on quadruplicate tests. Different letters indicate significant differences between treatments within the same parameter (*p* < 0.05), based on one-way ANOVA followed by Tukey’s post hoc test when parametric assumptions were met (for: **D**,**F**,**H**), or Kruskal–Wallis followed by Dunn’s post hoc test when assumptions were not met (for: **A**–**C**,**E**,**G**).

In the first experiment, fresh duckweed was ensiled with and without silage corn, amongst other conditions, to evaluate its potential for silage conservation (Table 1; Test I), and the results are shown in Figure 1 and in Appendix A: Table A3 and Table A4.

The pH of pure duckweed silages (T2 and T3) decreased only marginally, stabilizing at 6.1–6.2 after six weeks (Figure 1A). This limited acidification demonstrates the failure in creating a low-pH environment needed to suppress undesirable microbial activity. This insufficient acidification can be attributed to the unfavourable composition of duckweed, as its low WSC content and high moisture levels (Figure 1B) hamper the fermentation process by lactic acid bacteria. The substantial fresh weight losses observed for the sole duckweed silages (Figure 1C) agree with the hypothesis of continuous microbial activity at higher pHs, with total fermentation losses reaching 11–16% after six weeks. These fermentation losses were substantially higher than for the reference with only silage corn (T1) and the mixtures of duckweed with silage corn (T4 and T5), which achieved pH values lower than 4. However, little significant differences with the reference silage (T1) and the mixed silages (T4 and T5) were detected for pH, dry matter content, or fermentation losses based on the non-parametric analyses applied, as the ANOVA assumptions were not met for these parameters (see Figure 1A–C).

When evaluating silage quality, the ammonia fraction is a key indicator in practice [9,21]. It reflects the proportion of nitrogen from crude protein that has been converted into ammonia nitrogen, serving as an indicator of protein degradation and overall preservation quality. Depending on the silage and moisture content, typical ammonia fractions range from 5 to 12% [21]. As presented in Figure 1D, pure duckweed silages showed ammonia fractions of 56–57%, which were significantly higher than those in the corn reference (T1) and the duckweed–corn mixtures (T4 and T5). These values clearly exceeded acceptable limits and further indicate poor preservation.

In Figure 1E–H, the lactic acid (LA) concentrations in the different mixtures are presented, together with the concentrations of other fermentation products like acetic acid (AA), butyric acid (BA), and ethanol (EtOH). The LA concentrations in the duckweed-only silages remained below the limit of quantification, which was expected as LA typically is the dominant acid in well-fermented silages, playing a key role in pH reduction during silage fermentation [21]. As a result of the insufficient acidification, undesirable microbial processes could take place. For example, clostridial organisms thrive under wet conditions (>70% moisture) and convert LA into BA, a process normally inhibited by low pH [21]. Accordingly, the BA concentrations in the silages with only duckweed were extremely high (54.6–56.0 g/kg DM). BA production is associated with significant digestible energy losses, reduced silage quality, and a rancid odour [21]. Additionally, a high concentration of BA in silage can cause issues in cheese production if milk from dairy cows fed with contaminated silage is used [21]. In practice, silages with BA concentrations below 2 g/kg DM are considered good, while concentrations exceeding 5 g/kg DM are deemed poor [9]. Therefore, the BA levels observed in the pure duckweed silages substantially exceed acceptable thresholds. Similar results were reported by Hoving et al. (2011)*,* who measured a BA concentration up to 45 g/kg DM in duckweed silage without additives [9].

For LA and BA, these substantial descriptive differences between treatments were not all detected as statistically significant (Figure 1E,G). However, the statistical outcome should be interpreted cautiously, because the assumptions for parametric testing were not met and non-parametric tests were therefore applied.

Acetic acid (AA) concentrations in the pure duckweed silages were also exceptionally high, i.e., around 160 g/kg DM (Figure 1F). While moderate AA levels (10–30 g/kg DM) can enhance aerobic stability due to their antifungal properties, excessive concentrations (>40–60 g/kg DM) are typically associated with wet silages experiencing undesirable fermentations dominated by enterobacteria, clostridia, or heterofermentative lactic acid bacteria [22]. Such conditions were likely present here, as indicated by the combined presence of high AA and ammonia nitrogen, featuring characteristics of clostridial fermentations [21].

Ethanol concentrations in these silages were similarly elevated, exceeding 60 g/kg DM (Figure 1H). Ethanol is commonly produced in silages by heterofermentative lactic acid bacteria, enterobacteria, and yeasts [21]. In well-preserved silages, ethanol levels typically remain between 5–15 g/kg DM. However, concentrations above 30–40 g/kg DM are often linked to high yeast activity, fermentation losses and result in silages that spoil rapidly upon air exposure [21].

On the contrary, when duckweed was co-ensiled with silage corn, all the evaluated parameters fell within the expected ranges for a well-preserved silage, indicating that the co-ensiling strategy is a promising one for preservation of duckweed for feed applications.

### 3.2. Washing Duckweed Had No Clear Effect on Silage Quality in Test I

As the duckweed used in Test I was cultivated in a system fertilised with pig manure effluent, which may contribute to microbial and organic contamination, the potential effect of washing duckweed prior to ensiling was evaluated (Test I, Table 1). Washing was intended to remove residual cultivation medium. Its effect was evaluated based on the measured silage quality parameters, including dry matter content, fermentation losses, protein content, pH, ammonia concentration, ammonia fraction, and fermentation products.

As presented in Figure 1, washing resulted in 1.4 times more fermentation losses compared to unwashed duckweed when ensiled without a co-substrate. Nevertheless, there was no notable difference in dry matter content or any of the other evaluated parameters between the treatments. Overall, washing duckweed before ensiling had no significant effect on any silage quality parameter. Both treatments resulted in poor silage characterized by unacceptably high losses and low quality, as discussed in the previous section.

In the same experiment, washed and unwashed duckweed were also mixed with silage corn to evaluate a co-silage strategy. Treatments T4 (unwashed duckweed with silage corn) and T5 (washed duckweed and silage corn) showed a reversed trend compared to the pure duckweed silages, as fermentation losses were slightly lower in the washed treatment (1.3 times less loss). As with the silages containing only duckweed, no significant differences were observed over the evaluated parameters. These results suggest that washing duckweed prior to ensiling did not consistently influence the measured silage quality parameters in Test I.

### 3.3. The Choice of Substrate Affects the Success of a Co-Ensiling Strategy

As discussed, mixing duckweed with silage corn improved silage quality substantially. In Test I, co-ensiling reduced fermentation losses by a factor of 20 and ammonia-N levels by a factor of 8 compared to pure duckweed silages (Figure 1). These mixtures were formulated to exceed 30% dry matter (DM), a threshold known to suppress clostridial fermentation. However, this implied limited duckweed inclusion to just 12.5%, requiring seven parts of silage corn per part of duckweed.

Therefore, Test II explored alternative co-substrates and additives that could support higher duckweed inclusion while still ensuring adequate silage preservation. Additionally, identifying alternative co-substrates is particularly valuable in regions or periods where/when fresh silage corn is not readily available near duckweed cultivation sites. Previously ensiled corn and grass silages were also included because they can be stored and used when needed as co-substrates. In total, nine starting materials, including a control containing only duckweed, were formulated to reach an intended DM content of approximately 30%. These included drier substrates such as beet pulp, hay, and hemp shives, as well as molasses as a fermentable sugar source in an attempt to enable the ensiling of pure duckweed. Combinations of fresh and sun-dried duckweed were also evaluated with this aim.

**Figure 2:**
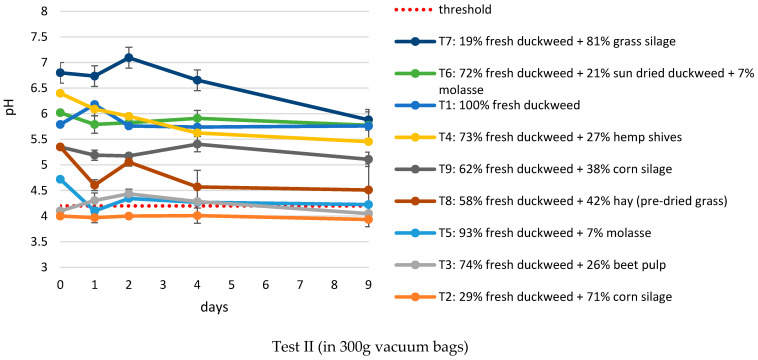
pH gradient of the 9 different starting materials tested in the screening Test (Test II, Table 1). Each measurement was performed in duplicate; the values shown represent the mean value, and standard deviations are indicated. A threshold value of 4.2 is indicated and is accepted in the literature as the minimum pH for a good (wet) silage.

Test II was designed as a short and low-labour screening test to compare a relatively large number of co-substrates and additives in one experimental run. Therefore, pH evolution was selected as the primary screening parameter. This choice was based on the central role of rapid acidification during the fermentation phase of ensiling [21]. After the initial aerobic phase, the silo becomes anaerobic and lactic acid bacteria produce lactic acid, resulting in a pH decline that inhibits undesirable microbial activity. If the pH does not decrease sufficiently during the first days of ensiling, successful long-term preservation is unlikely. Day 9 was selected as the final screening point because it allowed evaluation of early acidification while limiting practical problems related to gas accumulation in the vacuum bags, which were not equipped with a gas-release system, like the microsilos in Tests I and III. However, pH alone does not fully describe silage quality, as it does not provide information on fermentation losses, ammonia formation or organic acid profiles. Therefore, Test II was used only to select promising candidates for further evaluation in Test III, rather than to confirm successful ensiling. The pH evolution over a nine-day ensiling period is shown in Figure 2.

Immediately after the preparation of the mixtures and prior to ensiling, the pH of the nine different starting materials was measured. These initial values are presented in Figure 2 at day 0 on the *x*-axis. There was a clear variation in the initial pH values among the starting materials. Notably, mixtures T5, T3, and T2 already exhibited a low pH (<5.0) before ensiling. For these treatments, particular attention was given to whether the pH remained sufficiently low during storage, ideally below the critical threshold of pH 4.2, which is widely supported by silage literature. Kung et al. (2018) reported optimal pH ranges of 4.3–4.7 for grass silages (25–35% moisture) and ≤4.3 for corn silages with >30% moisture [21]. McDonald et al. (1991) noted that final silage pH depends on the buffering capacity of the substrate, with corn silage typically achieving lower pH values (3.7–4.0) due to its low buffering capacity, while legumes may stabilize at higher pH levels (4.3–5.0) [22]. Hoving et al. (2011) highlighted the need for lower final pH in silages with higher moisture content [9].

Treatments T5, T3, and T2 remained below the pH threshold of 4.2 throughout the ensiling period and were therefore selected as promising candidates for the subsequent, more in-depth third ensiling experiment. The fact that T5 maintained a sufficiently low pH was surprising, given its low dry weight content (11%), which typically poses a risk for poor silage fermentation. On the other hand, mixture T6 showed less acidification, despite having a higher dry matter content due to the inclusion of sun-dried duckweed. Higher dry matter content is generally associated with more favourable conditions for acidification. A possible explanation is that sun-drying may have affected the naturally occurring microbial community of the duckweed and thereby reduced spontaneous lactic acid fermentation. However, this was not directly assessed and should therefore be considered only as a hypothesis.

For the starting materials with higher initial pH values, the focus was primarily on the extent of acidification during the 9-day ensiling period. Notably, treatments T4 and T7, containing hemp shives and grass silage, respectively, exhibited promising pH declines. Since grass silage is generally more readily available than hemp shives as a co-substrate, it was selected for in-depth investigation in the subsequent ensiling experiment.

### 3.4. Corn Silage as a Co-Substrate Offered the Most Stable Fermentation Profile, Closely Followed by Grass Silage

**Figure 3:**
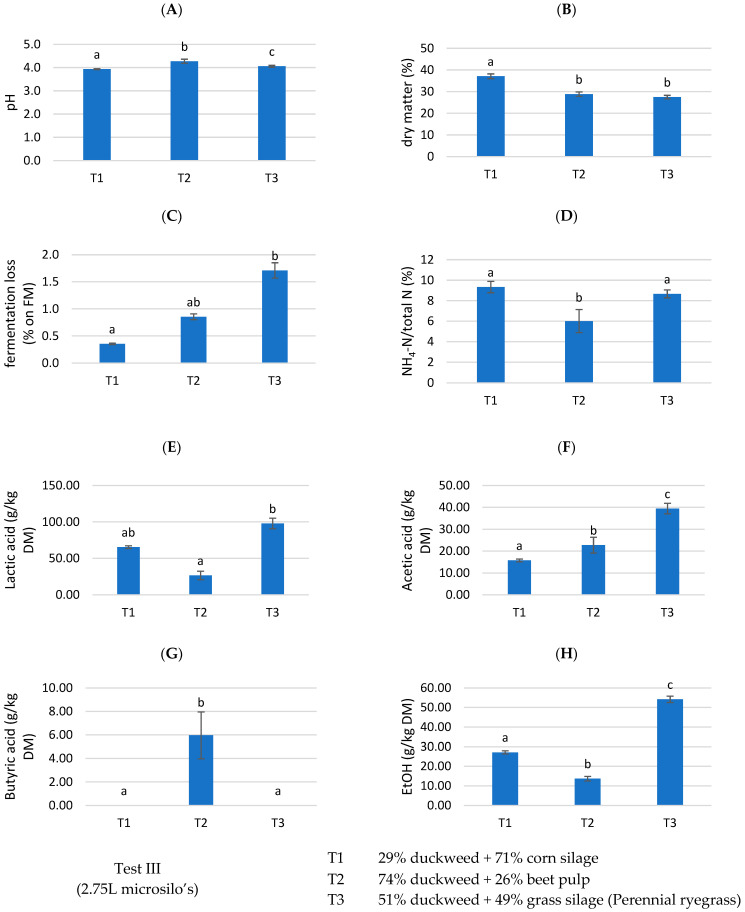
pH (**A**), dry matter content (**B**), total fermentation loss (**C**), ammonia fraction (**D**), Lactic acid (**E**), acetic acid (**F**), butyric acid (**G**), and ethanol concentration (**H**) on dry matter basis, measured on 3 different silage mixtures after a 102-day ensiling period (Test III, Table 1 and as presented in the legend). Mean values are presented with standard deviations, based on quadruplicate tests. Different letters indicate significant differences between treatments within the same parameter (*p* < 0.05), based on one-way ANOVA followed by Tukey’s post hoc test when parametric assumptions were met (for: **C**,**E**,**G**) or Kruskal–Wallis followed by Dunn’s post hoc test when assumptions were not met (for: **A**,**B**,**D**,**F**,**H**).

In ensiling Test III, four starting materials were prepared based on the results from the pH-screening experiment (Test II, Table 1). This follow-up test focused on duckweed co-ensiled with corn silage, grass silage, beet pulp, and molasses as co-substrates. For the duckweed–molasses mixture, limestone powder (CaCO_3_) was added to absorb excess moisture and increase the dry matter content; a technique based on prior silage research with fodder beets [23]. This treatment was selected because the molasses-based mixture showed promising pH evolution during Test II. However, in the long-term experiment, the mixture remained too wet and fluid to be properly stored in the microsilos for the planned 102-day ensiling period, despite the addition of CaCO_3_. It was therefore vacuum-sealed in a plastic bag, as in Test II, but excessive gas formation occurred during the first week, leading to its removal from the experiment. This was likely more problematic in Test III because a larger amount of material was packed per vacuum bag than in the short screening test [23]. As a result, only three treatments were fully evaluated in Test III: T1 (duckweed–corn silage), T2 (duckweed–beet pulp), and T3 (duckweed–grass silage). Key indicators of silage fermentation performance, i.e., fermentation losses, dry matter content, pH, and ammonia fraction, are displayed in Figure 3A–D, together with the concentrations of fermentation products in Figure 3E–H. The composition of the starting materials is shown in Appendix A Table A4, and all the measured fermentation quality parameters are presented in Table A1 and Table A5.

The pH of all three silages decreased to an acceptable level, with T1 reaching the lowest pH. This is consistent with its higher dry matter content and with literature reports indicating that corn silages typically achieve lower final pH values than grass silages due to their lower buffering capacity [21]. In contrast, T2 showed the least acidification, which may indicate reduced silage stability. The trend in pH and dry matter content was also reflected in the fermentation losses, which differed substantially among treatments. T1 exhibited the lowest losses, followed by T2 and then T3. Nevertheless, fermentation losses remained relatively limited across all three mixtures.

The crude protein contents also differed between treatments (see Section 3.6 or Appendix A Table A4). This mainly reflected the different co-substrates and duckweed inclusion rates, with T1 having the lowest crude protein content and T2 and T3 having higher values both before and after ensiling. Regarding protein degradation during ensiling, as inferred from the ammonia fraction, T2 performed significantly better than both T1 and T3, but all within an acceptable range [21]. These differences in protein content may have contributed to the observed fermentation profiles, as higher silage protein levels have been associated with increased pH, acetate formation, ethanol concentration, and proteolysis [24]. However, these effects cannot be attributed to protein content alone, because the treatments also differed in dry matter content, co-substrate type, and duckweed inclusion rate.

T2 demonstrated good protein preservation, but showed clear signs of clostridial fermentation. It contained an average of 5.96 g butyric acid/kg DM, whereas no butyric acid was detected in T1 or T3. According to Kung et al. (2018), butyric acid should be absent in well-fermented silages, as its presence signals clostridial activity associated with dry matter losses, energy degradation, and reduced palatability [21]. T2 also exhibited the lowest lactic acid content (26.57 ± 3.61 g/kg DM), the highest pH (4.27), and an unfavourable lactic-to-acetic acid ratio (LA/AA, 1.22 ± 0.46), indicating poorer fermentation stability.

In contrast, T1 showed the most favourable silage fermentation characteristics. It achieved a strong lactic-to-acetic acid ratio (LA/AA = 4.16 ± 0.15), no detectable butyric acid, a low pH (3.94 ± 0.02), and minimal fermentation losses. However, it had a relatively high ammonia fraction, but this may be attributable to its lower initial protein content rather than to poor silage preservation. T3 also achieved high lactic acid production (97.90 ± 7.22 g/kg DM) with no butyric acid, but showed significantly higher acetic acid and ethanol concentrations (resp. 39.47 ± 2.40 and 54.18 ± 1.59 g/kg dm), suggesting a less stable fermentation. Moderate acetic acid formation may improve aerobic stability by inhibiting yeasts, while high acetic acid concentrations (>5–6%) are often detected in very wet mixtures (>70% moisture) and are typically associated with undesirable fermentations dominated by enterobacteria or clostridia [22]. Elevated ethanol concentrations (>3–4%) are generally associated with yeast activity, and such silages usually spoil readily when exposed to air because some yeasts can assimilate LA under these conditions [21].

Overall, corn silage as a co-substrate offered the most stable and uncontaminated fermentation profile, with low pH, limited fermentation losses, absence of butyric acid and a favourable LA/AA ratio. However, this stability was achieved at a relatively low duckweed inclusion rate, as a high proportion of corn silage was needed to increase dry matter content and support fermentation. In contrast, beet pulp enabled the highest duckweed inclusion and showed the best protein preservation, but the tested formulation was susceptible to clostridial spoilage, likely due to its lower dry matter content. This highlights an important practical trade-off between fermentation stability and duckweed inclusion rate. With further formulation optimization, for example by increasing the beet pulp fraction or applying lactic acid bacteria inoculants, beet pulp could become a particularly relevant co-substrate for duckweed storage, especially because it is available as dried, easily storable pellets. Grass silage represented an intermediate strategy, allowing a higher duckweed inclusion rate than corn silage while maintaining a more stable fermentation profile than the beet pulp mixture under the tested conditions.

The ability to use different crops for co-ensiling throughout the growing season offers flexibility, enabling duckweed to be preserved with locally and seasonally available substrates. Moreover, the fact that pre-ensiled corn and grass silages were successfully re-ensiled with duckweed further increases the practical availability of these substrates, as they can be stored until the moment they are needed for mixing with duckweed.

The practical handling of duckweed-based silages at larger scale remains an important point for further research. This includes the optimisation of mixing duckweed with the co-substrates, silo type, compaction, and effluent management. Hoving et al. already reported that conventional bale wrapping is difficult for duckweed because of its low structural content and high moisture content [9]. Depending on the co-substrate and duckweed inclusion rate, this may need to be reconsidered for mixed silages with improved structure and dry matter content. In practice, sufficient dry co-substrate should be added to avoid effluent juice losses and associated handling requirements.

### 3.5. Ensiling Reduces Oxalic Acid in Duckweed–Grass Mixtures

**Figure 4:**
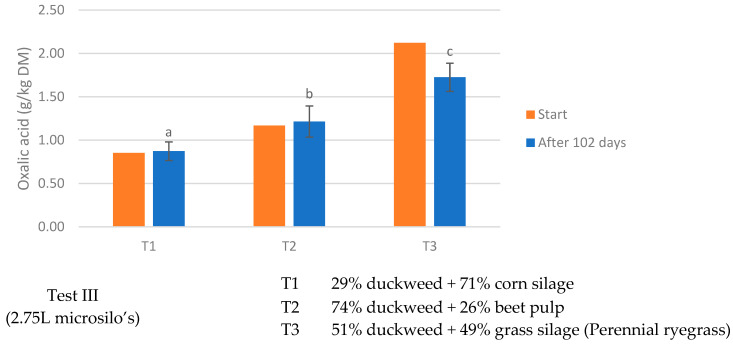
Oxalic acid content in g/kg dry weight of T1 (duckweed + corn silage), T2 (duckweed + beet pulp, and T3 (duckweed + grass silage) before and after an ensiling period of 102 days. Mean values are presented with standard deviations, based on quadruplicate tests. Different letters indicate significant differences between treatments after ensiling (4n) within the same parameter (*p* < 0.05), based on one-way ANOVA followed by Tukey’s post hoc test (parametric assumptions were met).

In addition to fermentation products such as lactic, acetic, propionic, and butyric acid, the oxalic acid concentrations were also measured before and after the 102 days of storage (Test III). Unlike the aforementioned acids, which are produced by microbial activity during ensiling, oxalic acid is primarily derived from the plant material itself. The goal was to assess whether the ensiling process influences oxalic acid concentrations in the tested mixtures (T1, T2, T3, see Table 1). A reduction in oxalic acid is considered beneficial, as it acts as an anti-nutritional factor by binding divalent minerals such as calcium and magnesium, thereby reducing their bioavailability in animal feed [25]. Figure 4 presents the oxalic acid concentrations in the mixtures before and after ensiling.

A clear reduction in oxalic acid content was observed only in the duckweed–grass silage (T3), suggesting that the fermentation process influenced its degradation in this mixture. The impact of ensiling on oxalic acid levels has been previously explored, but findings vary across studies, as observed in our results. For example, Abbasi et al. (2018) reported no change in oxalic acid concentration after ensiling of Amaranthus hypochondriaus [26]. Similarly, Udén (2018) found no difference in the oxalic acid content of corn silages before and after silage fermentation [27], whereas Martens et al. (2014) found substantial reductions (51–100%) when ensiling tropical forage legumes [28].

In their study, the most significant reductions occurred when silages were inoculated with *Lactobacillus plantarum* and supplemented with sucrose, leading to complete oxalic acid degradation [28]. This effect has been linked to the oxalate-degrading capacity of certain lactic acid bacteria strains, as described by Turroni et al. (2007) [29]. In our experiment, the duckweed–grass silage (T3) showed both the highest sugar content and lactic acid concentration (see Table A2 and Table A4), supporting the findings of Martens et al. (2014) that enhanced lactic fermentation can promote oxalic acid breakdown [28]. These results suggest that targeted fermentation conditions may improve the nutritional quality of duckweed-based silages by reducing anti-nutritional compounds like oxalic acid.

However, it is important to note that the oxalic acid concentrations observed across all three mixtures were relatively low and do not pose a concern from a feed safety perspective. For fresh *Lemna × japonica* 6580 an oxalic acid concentration of 0.26 g/kg FW was measured, which can be considered low compared to other concentrations reported for *Lemna minor* and *Lemna gibba*, ranging between 0.7 and 1.1 g/kg FM [30]. At these levels, oxalic acid is unlikely to limit the use of duckweed-containing silages in animal diets. This conclusion is supported by comparisons with much higher silage concentrations reported by Martens et al. (up to tenfold greater) [28], as well as by typical values found in various food and feed crops, such as beetroot (up to 6.8 g/kg FW), spinach (7 g/kg FW), rhubarb (8 g/kg FW), and brussels sprouts (15 g/kg FW) [31,32].

### 3.6. Duckweed–Grass Mixtures Retain the Highest Level of Pepsin-Digestible Protein After Ensiling

Ensiling led to a reduction in total protein content across all the starting materials, which can be attributed to proteolysis and fermentation-related nitrogen losses. Proteolysis breaks down proteins into peptides, amino acids, and nitrogenous end-products like ammonia, which can reduce the nutritional value of the silage. Less is known, however, about the effect of ensiling on the pepsin digestibility of proteins. This parameter provides an estimate of gastric protein digestibility, which is particularly relevant for monogastric animals.

**Figure 5:**
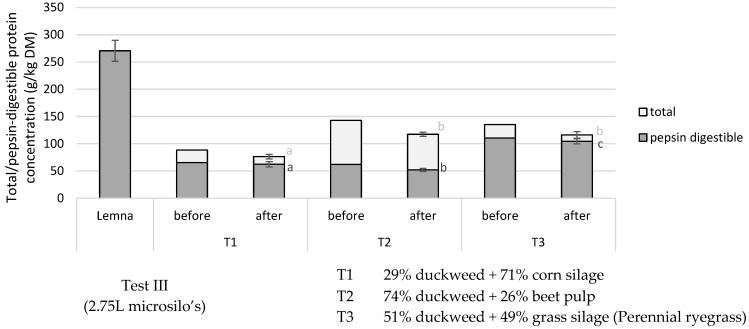
Total and pepsin-digestible protein content on dry matter measured for T1 (duckweed + corn silage), T2 (duckweed + beet pulp, and T3 (duckweed + grass silage) before and after a 102-day ensiling period. Mean values are presented with standard deviations, based on quadruplicate tests. Different letters indicate significant differences between treatments after ensiling (4n) within the same parameter (*p* < 0.05), based on one-way ANOVA followed by Tukey’s post hoc test (parametric assumptions were met).

In Test III, total protein content and pepsin digestibility of the three mixtures (T1: duckweed–corn, T2: duckweed–beet pulp, T3: duckweed–grass) were assessed before ensiling (one sample, analysed in duplicates) and after an ensiling period of 102 days (four biological replicates, analysed in singlet). As shown in Figure 5, pepsin digestibility remained relatively stable across all the treatments, with T1 and T3 showing slight improvements. After ensiling, 11% and 10% more of the total protein in T1 and T3, respectively, was pepsin-digestible compared to pre-ensiling levels.

The duckweed–beet pulp mixture (T2) initially had the highest crude protein content after ensiling. Significantly higher than the duckweed–corn mixture (T1). However, due to its low pepsin digestibility (44%), it retained a significantly lower digestible protein content after ensiling compared to the other two mixtures. The duckweed–corn mixture (T1), despite its lower initial protein content, had a much higher digestibility (82%), resulting in greater protein availability post-ensiling. After the 102-day ensiling period, T2 and T3 exhibited comparable total protein levels, but T3 retained a significantly higher proportion of digestible protein. Among the three treatments, the duckweed–grass mixture (T3) offered the best combination of protein content and digestibility, followed by T1. The duckweed–beet pulp silage (T2) showed the poorest performance in terms of retaining digestible protein.

The crude protein and pepsin-digestible protein content of *Lemna* used in the mixtures were also measured. The crude protein content was 26.6% on DM, and the apparent pepsin-digestible protein content was 27.1 ± 1.9% on DM, suggesting a high apparent pepsin-digestible protein fraction under the applied conditions. This indicates that the differences in apparent pepsin-digestible protein observed between the mixtures were likely influenced by the co-substrates and their inclusion rates, rather than by the *Lemna* fraction itself. However, this result should be interpreted cautiously because crude protein was measured according to ISO 16634-1 using Kjeldahl nitrogen determination (with factor 6.25) [12], whereas pepsin-digestible protein was quantified in the supernatant using the Lowry method with BSA as standard. These methodological differences may have led to an overestimation of the apparent pepsin-digestible protein fraction.

No direct literature data on the pepsin digestibility of *Lemna* protein are available for comparison. However, several in vitro digestibility studies have been conducted using combined pepsin and pancreatin digestion, simulating both gastric and small intestinal conditions. These studies reported high in vitro digestibility values of 72% and 75.4% for *Lemna* with crude protein contents of 22.2% and 25.5% on DM, respectively [33,34]. Compared to these findings, the results from the present study suggest that *Lemna* protein is mainly degraded in the small intestine.

Direct drying could be an alternative preservation route, as it further concentrates duckweed biomass and increases protein content on a fresh weight basis. However, drying also concentrates minerals and other compounds, which may be beneficial or limiting depending on the final feed formulation and inclusion level. Moreover, drying fresh duckweed is challenging because of its very high moisture content and may require substantial energy input. The economic feasibility of drying is likely location-dependent; in warmer climates, sun-drying may offer a more feasible low-cost option.

### 3.7. Microbiological Evaluation of the Duckweed–Beet Pulp Silage Indicates Improved Enterobacteriaceae Control but Increased Clostridial Risk

Microbiological analysis is essential when evaluating novel feed ingredients such as duckweed, especially when grown on waste streams. Such analysis not only ensures compliance with feed safety standards but also provides insight into the microbial stability of the resulting silage. In this study, microbiological testing was performed on the fresh duckweed used in Test III and on the duckweed–beet pulp silage (T2) obtained after an ensiling period of 102 days. These samples were selected to represent a worst-case scenario due to the high proportion of duckweed in T2. Moreover, as discussed in Section 3.4, T2 showed indications of clostridial activity based on elevated butyric acid concentrations. This was further investigated here through the quantification of sulphite-reducing anaerobes, enabling a direct comparison between fresh and ensiled biomass. A full overview of the measured parameters and results is provided in Appendix A, Table A3.

According to Belgian legislation, feed materials must be free of *Salmonella* and contain fewer than 300 CFU/g FW of *Enterobacteriaceae*. In addition, non-mandatory but commonly applied standards such as the Feed Chain Alliance (FCA) guidelines include limits for *Clostridia* (<1000 CFU/g FW) and fungi (<10,000 CFU/g FW). Some export markets further impose thresholds on aerobic colony counts (ACC) at 30 °C.

No *Salmonella* was detected in either the fresh or ensiled samples. While fresh duckweed exceeded the legal limit for *Enterobacteriaceae* in all the replicates, ensiling with beet pulp resulted in a marked reduction to below detectable levels, meeting Belgian legal standards. However, ensiling led to a substantial increase in *Clostridia* counts, exceeding the FCA threshold in all the replicates of T2. It should be noted that T2 also showed the highest butyric acid concentrations and final pH (Section 3.4), suggesting less efficient acidification and a higher likelihood of clostridial activity. Duckweed mixtures T1 and T3 did not exhibit elevated butyric acid levels and may therefore present a lower clostridial risk under similar ensiling conditions; however, this was not confirmed microbiologically. Fungal counts decreased post-ensiling, while ACC values increased by approximately two orders of magnitude.

These findings suggest that ensiling duckweed may improve microbiological safety by reducing *Enterobacteriaceae*. However, further microbiological testing with other co-substrates is needed to generalise this statement for all the duckweed silage mixtures. Additionally, the risk of clostridial fermentation remains, particularly when silage conditions do not support rapid and sufficient acidification. As previously noted, higher dry matter content, the use of sugar-rich co-substrates, and/or homofermentative lactic acid bacteria containing inoculants could mitigate this risk.

While *C. botulinum* was not specifically assessed in this study, previous research by Hoving et al. (2011) reported its presence in fresh duckweed, which was suppressed effectively through the use of silage additives [9]. High-moisture forages with slow acidification are especially susceptible to colonization by *Clostridium tyrobutyricum*, known for producing butyric acid and biogenic amines, which can negatively affect feed intake, health, and milk quality.

Chemical contaminants were not analysed in the present ensiling study. However, for duckweed grown on wastewater-derived media, feed application requires additional safety assessment for contaminants such as heavy metals or other micropollutants, as also considered in previous work on duckweed cultivation on pig manure-derived streams [1,3].

## 4. Conclusions

This study demonstrates that duckweed can be preserved and valorised as a feed ingredient through co-ensiling with suitable agricultural co-substrates. Among the tested mixtures, corn silage resulted in the most stable fermentation profile, with low pH, limited fermentation losses, absence of butyric acid, and a favourable lactic-to-acetic acid ratio. Grass silage allowed a higher duckweed inclusion rate and retained the highest level of apparent pepsin-digestible protein after storage, but its relatively high acetic acid and ethanol concentrations indicate that further optimisation is needed to improve fermentation stability. The beet pulp mixture allowed the highest duckweed inclusion rate, but exhibited poor stability, with signs of clostridial fermentation, likely due to excessive moisture. Microbiological analysis of this duckweed–beet pulp mixture showed reduced Enterobacteriaceae counts after ensiling, but also confirmed increased clostridial risk under suboptimal conditions. Oxalic acid concentrations were low in all the duckweed-based silages, and the largest reduction during storage was observed in the duckweed–grass mixture. The fresh duckweed used in this study showed a high apparent pepsin-digestible protein fraction, although this should be interpreted cautiously because of methodological differences between crude protein and pepsin-digestible protein determination. Overall, co-ensiling duckweed with corn silage, grass silage, or optimised beet pulp formulations offers a promising strategy to produce storable and nutritionally valuable feedstuffs, provided that dry matter content and fermentation conditions are sufficiently controlled.

## Figures and Tables

**Table 1:** Overview of the tested starting materials in the three experiments, which used duckweed from a pig manure treatment system (Test I) or an aquaculture treatment system (Test II and III). The composition of the tested starting materials is given in mass percentages, and the dry matter content prior to ensiling is given in the last column.

Test	Replicates	Starting Materials (m%)		DM (%)
I	4	T1	100% silage corn *	39
	4	T2	100% duckweed	6
	4	T3	100% washed duckweed	6
	4	T4	12.5% duckweed + 87.5% silage corn *	32
	4	T5	12.5% washed duckweed + 87.5% silage corn *	33
II	2	T1	100% duckweed	8
	2	T2	29% duckweed + 71% corn silage	29
	2	T3	74% duckweed + 26% beet pulp	30
	2	T4	73% duckweed + 27% hemp shives	30
	2	T5	93% duckweed + 7% molasses	11
	2	T6	72% duckweed + 21% sun-dried duckweed + 7% molasses	31
	2	T7	19% duckweed + 81% grass silage (Perennial ryegrass)	21
	2	T8	58% duckweed + 42% hay (heavily prewilted grass)	21
	2	T9	62% duckweed + 38% corn silage	22
III	4	T1	29% duckweed + 71% corn silage	40
	4	T2	74% duckweed + 26% beet pulp	30
	4	T3	51% duckweed + 49% grass silage (Perennial ryegrass)	32
	4	T4	74% duckweed + 7% molasses + 19% CaCO_3_	30 **

* In experiment I, fresh corn was used; in experiments II and III, a corn silage was opened and ensiled again as co-substrate. Whole plant corn was always used. ** Calculated based on the measured dry matter contents of the individual components before mixing and the intended mixture composition; the final mixed material was not analysed separately because this treatment was excluded from the long-term evaluation.

## Data Availability

The data supporting the findings of this study will be made available on Zenodo after publication using the same title as the manuscript to facilitate retrieval. Additional information can be obtained from the corresponding author upon request.

## Appendix A

**Table A1:** The average dry matter contents (DM), density, fermentation losses, water-soluble carbohydrates (WSC), protein content, pH, NH_3_ content and NH_3_ fraction of the first and third series of ensiling experiments as described in Table 1, measured after an ensiling period of resp. 42 and 102 days. (-) indicates that this value was not measured.

		DM (%)	Density	Fermentation Losses	WSC	Protein Content	pH	NH3	NH3 Fraction
Test		(%)	(kg DM/m^3^)	(% on FW)	(g/kg DM)	(g/kg DM)		(g/kg DM)	(%)
I	T1	39.45 ± 1.28	191.95 − 1.79	0.57 ± 0.34	-	78.94 ± 1.36	3.90 ± 0.13	0.48 ± 0.04	3.02 ± 0.26
	T2	5.81 ± 0.07	49.01 − 0.11	11.16 ± 1.43	-	253.87 ± 13.42	6.24 ± 0.05	66.45 ± 1.72	57.41 ± 1.23
	T3	5.67 ± 0.08	46.5 − 0.29	15.92 ± 2.30	-	273.28 ± 9.91	6.14 ± 0.07	68.53 ± 1.01	56.36 ± 1.12
	T4	31.35 ± 0.40	191.64 − 0.17	0.56 ± 0.15	-	102.28 ± 2.20	4.09 ± 0.34	1.50 ± 0.19	7.01 ± 0.71
	T5	32.03 ± 0.46	195.37 − 0.19	0.43 ± 0.05	-	102.59 ± 2.43	3.97 ± 0.02	1.45 ± 0.17	6.78 ± 0.68
III	T1	37.10 ± 1.10	226.61 ± 0.36	0.35 ± 0.01	<5.25	76.34 ± 4.79	3.94 ± 0.02	1.38 ± 0.09	9.33 ± 0.56
	T2	28.83 ± 0.99	167.38 ± 0.16	0.86 ± 0.05	<5.25	117.30 ± 2.79	4.27 ± 0.09	1.37 ± 0.27	6.01 ± 1.13
	T3	27.47 ± 0.81	189.48 ± 1.86	1.71 ± 0.14	6.17	116.10 ± 4.74	4.06 ± 0.04	1.96 ± 0.09	8.67 ± 0.38

**Table A2:** The dry matter (DM) content of the duckweed biomass, co-substrates, and additives used in the three ensiling tests.

Test	Co-Substrate/Additive Used	Dry Matter Content (%)
I	Duckweed	6
	Silage corn (fresh silage maize)	39
II	Duckweed	8
	Corn silage	39
	Beet pulp pellets	90
	Hemp shives	89
	Sun-dried duckweed	92
	Molasses	74
	Grass silage	24
	heavily prewilted grass	57
III	Duckweed	9
	Corn silage	52
	Grass silage	52
	Beet pulp pellets	90
	Molasses	74

**Table A3:** Results obtained after microbiological analyses, following the indicated method or calculation, performed on fresh duckweed ensiled with beet pulp in silage Test III and on the silage mixture T2 (duckweed and beet pulp, see Table 1) measured after an ensiling period of 102 days. (est.): amount quantified by estimation.

Parameter	Fresh Duckweed				Silage T2, Test III (see Table 1)				Unit	Method/Calculation
	1	2	3	4	1	2	3	4		
Detection of *Listeria* spp.	Absent	Absent	Absent	absent	absent	absent	absent	absent	Detection/25g FW	ISO 11290-1/Amd 1 [35]
Detection of Listeria monocytogenes	Absent	Absent	Absent	absent	absent	absent	absent	absent	Detection/25g FW	ISO 11290-1/Amd 1 [35]
Counting of Escherichia coli at 44 °C	2.7 × 10 (est.)	<10	<10	9.1 × 10 (est.)	<10	<10	<10	<10	cfu/g FW	Rapid’*E. coli* 2—AFNOR BRD-07/01-07/93
Counting of lactic acid bacteria at 30 °C	9.1 × 10	8.2 × 10^2^	2.3 × 10^3^	1.4 × 10^3^	1.9 × 10^8^ (est.)	2.9 × 10^8^ (est.)	2.2 × 10^8^ (est.)	1.9 × 10^8^ (est.)	cfu/g FW	ISO 15214 [36]
Counting of Enterobacteriaceae at 37 °C	7.1 × 10^4^	4.8 × 10^4^	8.5 × 10^4^	9.8 × 10^4^	<10	<10	<10	<10	cfu/g FW	ISO 21528-2 [37]
Counting of anaerobic spores at 37 °C	2.5 × 10^4^	2.9 × 10^4^	4.2 × 10^4^	>1.5 × 10^7^	1.6 × 10^7^	9.1 × 10^6^	2.4 × 10^7^	9.3 × 10^6^	cfu/g FW	SP-VG M005
Counting of yeasts at 25 °C	2.0 × 10^3^ (est.)	8 × 10^2^ (est.)	3.6 × 10^3^	8.2 × 10^3^ (est.)	9.1 × 10	1.4 × 10^2^	4.6 × 10	3.7 × 10^3^	cfu/g FW	ISO 7954 (1987) [38]
Counting of molds at 25 °C	1.3 × 10^4^	9.1 × 10^3^ (est.)	3.4 × 10^3^	5.5 × 10^3^	1.7 × 10^2^	9.1 (est.)	9.1 (est.)	9.1 (est.)	cfu/g FW	ISO 7954 (1987) [38]
Counting of aerobic spores at 30 °C	7.3 × 10^6^	8.5 × 10^6^	8.4 × 10^6^	7.5 × 10^6^	3.4 × 10^8^ (est.)	2.1 × 10^8^	2.9 × 10^8^	2.1 × 10^8^	cfu/g FW	ISO 4833 (part 1) [39]
Detection of Salmonella	absent	absent	absent	absent	absent	absent	absent	absent	Detection/25g FW	ISO 6579 [40]
Number of sulfite-reducing anaerobes at 37 °C (clostridia)	3.1 × 10^2^	2.5 × 10^2^	3.7 × 10^2^	3.5 × 10^2^	7.7 × 104	1.9 × 10^3^	6.6 × 10^3^	6.3 × 10^4^	cfu/g FW	AFNOR XP V 08-061
Presumed count of Bacillus cereus at 30 °C	3.6 × 10 (est.)	9.1 × 10 (est.)	1.6 × 10^2^	2.7 × 10^2^	9.1 (est.)	1.3 × 10^2^	6.4 × 10	1.4 × 10^2^	cfu/g FW	ISO 7932 [41]
Counting of coliform bacteria at 30 °C	8.2 × 10^4^	6.3 × 10^4^	8.5 × 10^4^	3.5 × 10^5^	<10	<10	<10	<10	cfu/g FW	ISO 4832 [42]
Detection of Staphylococcus aureus	detected	Absent	absent	absent	absent	absent	absent	absent	Detection/10g FW	Based on ISO 6888-3:2003(en) [43]

**Table A4:** The dry matter (DM), water-soluble carbohydrates (WSC) on dry matter and fresh weight, crude ash, crude protein content and the buffering capacity (expressed as HCl eq. and Lactic acid (LA) eq.) of the starting materials of Test I and III, before ensiling. (-) indicates that this value was not measured.

		DM	WSC		Crude Ash	Crude Protein	Buffering Capacity	
Test		(%)	(g/kg DM)	(g/kg FW)	(g/kg DM)	(g/kg DM)	(meq. HCl/kg FW)	(meq. LA/kg FW)
I	T1	39.3	-	-	-	-	-	-
	T2	6.3	-	-	-	-	-	-
	T3	6.0	-	-	-	-	-	-
	T4	31.9	-	-	-	-	-	-
	T5	32.6	-	-	-	-	-	-
III	Used duckweed	9.0	-	-	-	266	-	-
	T1	39.6	<5.5	<2.2	61	88	75.0	128.3
	T2	30.2	36.9	11.2	133	143	14.9	25.5
	T3	31.6	118.7	37.5	102	135	34.1	58.3

**Table A5:** The average lactic acid, acetic acid, butyric acid, propionic acid, oxalic acid, ethanol contents, the lactic acid–acetic acid ratio and the sum of all fermentation products of the first and third series of silage experiments as described in Table 1, measured after an ensiled period of resp. 42 and 102 days. (-) indicates that this value was not measured.

		Lactic Acid	Acetic Acid	Butyric Acid	Propionic Acid	Oxalic Acid	Alc	LA/AA	Sum of Fermentation Products
Test		(g/kg DM)	(g/kg DM)	(g/kg DM)	(g/kg DM)	(g/kg DM)	(g/kg DM)	(-)	(g/kg DM)
I	T1	42.42 ± 6.05	12.31 ± 1.02	0.00 ± 0.00	-	-	11.50 ± 2.31	3.44 ± 0.32	-
	T2	0.00 ± 0.00	159.91 ± 8.26	56.00 ± 6.73	-	-	67.89 ± 0.85	0.00 ± 0.00	-
	T3	0.00 ± 0.00	163.22 ± 7.34	54.65 ± 3.84	-	-	69.58 ± 0.94	0.00 ± 0.00	-
	T4	36.56 ± 21.37	16.60 ± 6.93	0.00 ± 0.00	-	-	14.80 ± 1.63	2.55 ± 1.46	-
	T5	48.27 ± 3.43	14.90 ± 2.55	0.00 ± 0.00	-	-	13.25 ± 1.32	3.32 ± 0.67	-
III	T1	65.47 ± 1.80	15.76 ± 0.64	0.00 ± 0.00	0.00 ± 0.00	0.87 ± 0.11	27.13 ± 0.79	4.16 ± 0.15	108.36 ± 2.62
	T2	26.57 ± 5.90	22.76 ± 3.61	5.96 ± 1.99	4.01 ± 1.28	1.22 ± 0.18	13.69 ± 1.15	1.22 ± 0.46	73.00 ± 1.88
	T3	97.90 ± 7.22	39.47 ± 2.40	0.00 ± 0.00	2.83 ± 0.56	1.73 ± 0.16	54.18 ± 1.59	2.48 ± 0.14	194.38 ± 7.15
